# A Phase II Study to Compare the Safety and Efficacy of Direct Oral Anticoagulants versus Subcutaneous Dalteparin for Cancer-Associated Venous Thromboembolism in Patients with Advanced Upper Gastrointestinal, Hepatobiliary and Pancreatic Cancer: PRIORITY

**DOI:** 10.3390/cancers14030559

**Published:** 2022-01-22

**Authors:** Jwa Hoon Kim, Changhoon Yoo, Seyoung Seo, Jae Ho Jeong, Baek-Yeol Ryoo, Kyu-pyo Kim, Jung Bok Lee, Keun-Wook Lee, Ji-Won Kim, Il-Hwan Kim, Myoungjoo Kang, Hyewon Ryu, Jaekyung Cheon, Sook Ryun Park

**Affiliations:** 1 Department of Oncology, Asan Medical Center, University of Ulsan College of Medicine, Seoul 05505, Korea; jhoonkim@korea.ac.kr (J.H.K.); yooc@amc.seoul.kr (C.Y.); syseo@amc.seoul.kr (S.S.); jaeho.jeong@amc.seoul.kr (J.H.J.); ryooby@amc.seoul.kr (B.-Y.R.); kkp1122@amc.seoul.kr (K.-p.K.); 2 Division of Oncology, Department of Internal Medicine, Korea University Anam Hospital, Korea University College of Medicine, Seoul 05505, Korea; 3 Department of Clinical Epidemiology and Biostatistics, Asan Medical Center, University of Ulsan College of Medicine, Seoul 05505, Korea; jungboklee@amc.seoul.kr; 4 Division of Hematology and Medical Oncology, Department of Internal Medicine, Seoul National University Bundang Hospital, Seoul National University College of Medicine, Seongnam 13620, Korea; imdoctor@snu.ac.kr (K.-W.L.); jiwonkim@snubh.org (J.-W.K.); 5 Department of Oncology, Haeundae Paik Hospital, Cancer Center, Inje University College of Medicine, Busan 47392, Korea; H00353@paik.ac.kr (I.-H.K.); daniel@paik.ac.kr (M.K.); 6 Division of Hematology and Oncology, Department of Internal Medicine, Chungnam National University Hospital, Chungnam National University College of Medicine, Daejeon 35015, Korea; hyewonryu@cnu.ac.kr; 7 Division of Hematology-Oncology, Department of Internal Medicine, Ulsan University Hospital, University of Ulsan College of Medicine, Ulsan 44033, Korea; 0733883@uuh.ulsan.kr

**Keywords:** dalteparin, rivaroxaban, apixaban, venous thromboembolism, gastrointestinal tract cancer, hepatobiliary cancer, pancreatic cancer

## Abstract

**Simple Summary:**

This prospective phase II trial evaluated the safety and efficacy of direct oral anticoagulants (DOACs) versus subcutaneous dalteparin for cancer-associated venous thromboembolism (CA-VTE) in patients with high-risk cancer types and currently active advanced cancers. The clinically relevant bleeding (CRB) as the primary endpoint and major bleeding (MB) more occurred in the DOAC group than in the dalteparin group, and the hazard ratio for CRB and MB was approximately three and four times more in the DOAC group than in the dalteparin group. Cancer involvement at the GI mucosa was also a significant risk factor for CRB. The extra caution is necessary when using DOAC therapy for CA-VTE in patients with advanced upper gastrointestinal (GI) tract, hepatobiliary, or pancreatic cancer.

**Abstract:**

Background: We evaluated the safety and efficacy of direct oral anticoagulants (DOACs) versus subcutaneous dalteparin for cancer-associated venous thromboembolism (CA-VTE) in patients with advanced upper gastrointestinal (GI) tract, hepatobiliary, or pancreatic cancer. Methods: This was a multicenter, randomized, open-label, phase II trial in five centers. Patients randomly received rivaroxaban (15 mg twice daily for 3 weeks, then 20 mg once daily)/apixaban (10 mg twice daily for the first 7 days, then 5 mg twice daily) or dalteparin (200 IU/kg once daily for the first month, then 150 IU/kg once daily). Randomization was stratified by the Eastern Cooperative Oncology Group Performance Status, primary cancer type, active chemotherapy, and participating centers. The primary endpoint was the rates of clinically relevant bleeding (CRB) in the full analysis set (FAS). Results: A total of 90 patients were randomly assigned to the DOAC (*n* = 44) and dalteparin groups (*n* = 46) in FAS. CRB and major bleeding (MB) rates were 34.1% and 13.0% (*p* = 0.018) and 18.2% and 4.3% (*p* = 0.047) for the DOAC and dalteparin groups, respectively. Time to CRB and MB was higher in the DOAC group than in the dalteparin group (hazard ratio [HR] 2.83; *p* = 0.031 and HR 4.32; *p* = 0.064). Cancer involvement at the GI mucosa was also a significant risk factor for CRB. Recurrent CA-VTE occurred in 2.3% and 2.2% of patients given DOAC and dalteparin, respectively (*p* = 1.000). Conclusion: DOAC therapy further increased the risk of bleeding compared with dalteparin in patients with active advanced upper GI tract, hepatobiliary, or pancreatic cancer, suggesting that extra caution should be taken when selecting anticoagulants for CA-VTE.

## 1. Introduction

Cancer-associated venous thromboembolism (CA-VTE) is a major and potentially life-threatening complication in cancer patients [1]. As cancer itself presents the risk of recurrence, aggravation, or bleeding during anticoagulation, balancing these risks is challenging when using anticoagulation therapy for CA-VTE [2,3]. Furthermore, tumor type, anticancer treatment, and patient organ function are associated with thrombosis and bleeding [4,5,6]. Cancer-specific studies to evaluate the efficacy and safety of anticoagulation therapy are necessary for optimal management of CA-VTE.

Low-molecular-weight heparin (LMWH), such as dalteparin, has been the standard treatment for CA-VTE, and it has better, or similar, efficacy and safety compared to vitamin K antagonists (warfarin) maintenance in cancer patients [7,8,9]. Following recent clinical studies comparing DOAC with LMWH, including the Hokusai VTE cancer [10], SELECT-D [11], Caravaggio [12], and ADAM-VTE [13] trials, the latest guidelines issued in 2020 by the National Comprehensive Cancer Network (NCCN) and American Society of Clinical Oncology (ASCO) [14] recommend direct oral anticoagulants (DOACs) such as edoxaban, rivaroxaban, and apixaban as treatment options equivalent to LMWH. However, concerns about the bleeding risk of DOAC therapy have been continuously raised. Furthermore, more caution is required when using DOAC therapy for gastrointestinal (GI) tract cancers, particularly upper GI cancers. The cancer-specific clinical trials comparing DOAC and LMWH included highly heterogeneous cancer types and patients without current cancer lesions during anticoagulation [10,11,12,13]. As such, the treatment outcomes, especially the safety of DOACs, need to be evaluated in patients with current active lesions and cancer types that pose a high bleeding risk. Our previous retrospective study focusing on advanced unresectable/metastatic upper GI or hepatopancreatobiliary cancer demonstrated that rivaroxaban was associated with increased risk of major bleeding (MB) (17.4% vs. 7.6%; *p* = 0.072) and clinically relevant bleeding (CRB) (31.9% vs. 14.3%; *p* = 0.019) compared with LMWH and high risk of VTE and bleeding [15]. Therefore, we aimed to prospectively evaluate the safety and efficacy of DOACs compared with those of dalteparin as a treatment for CA-VTE in patients with currently active and advanced upper GI tract, hepatobiliary, and pancreatic cancer.

## 2. Material and Methods

### 2.1. Study Design and Participants

This study was a multicenter, open-label, randomized, controlled phase II trial conducted across five institutions in Korea. Inclusion criteria included patients aged ≥19 and <80 years old, with histologically confirmed, advanced upper GI tract, hepatobiliary, and pancreatic cancer, and newly diagnosed (within 2 weeks before randomization) symptomatic or incidental lower extremity or upper extremity (jugular, innominate, subclavian, axillary, brachial) deep vein thrombosis (DVT) or pulmonary thromboembolism (PTE). VTE was documented by computed tomography (CT), ultrasonography, or perfusion scan. Advanced cancer indicated a currently active localized unresectable disease (nonmetastatic) and metastatic disease, which was subject for anticancer treatment. Diagnostic criteria of VTE are listed in Supplementary Table S1. Adequate coagulation (platelet count ≥75,000/μL, prothrombin time/international normalized ratio ≤2, activated partial thromboplastin time ≤1.5 × the upper limit of normal (ULN)), hepatic (aspartate aminotransferase or alanine aminotransferase ≤3 × ULN (≤5 × ULN in the presence of liver metastases)), and renal function (calculated creatinine clearance ≥30 mL/min) were required. The major exclusion criteria included taking anticoagulant therapy for more than three days prior to randomization, any dose of thrombolysis therapy, continuous antiplatelet agent use, a history of VTE, hemodynamic unstable PTE, current active bleeding or uncontrolled bleeding history within 4 weeks before randomization, liver cirrhosis (Child–Pugh score ≥ 7), uncontrolled hypertension, and brain metastasis.

This study was approved by the institutional review board of each participating center and conducted in accordance with the Declaration of Helsinki and Good Clinical Practice. All patients provided written informed consent. This trial was registered on http://www.clinicaltrials.gov (accessed on 4 May 2017) with the identifier NCT03139487.

### 2.2. Randomization and Trial Intervention

Eligible patients were randomly assigned (1:1) to the DOAC (rivaroxaban or apixaban) or dalteparin group. Randomization was centrally performed through an interactive online system and stratified according to the Eastern Cooperative Oncology Group Performance Status (ECOG PS 0–1 vs. ≥2), primary cancer type (GI tract cancer vs. others), active chemotherapy (yes vs. no), and participating centers (Asan Medical Center vs. others). In the DOAC group, the choice of either rivaroxaban or apixaban was at the discretion of the treating physician.

DOACs (rivaroxaban or apixaban) or dalteparin were administered according to standard dosing schedules: 15 mg rivaroxaban was administered orally twice daily for the first 21 days, followed by 20 mg once daily; 10 mg apixaban was administered orally twice daily for the first 7 days and 5 mg twice daily thereafter; and 200 IU/kg dalteparin was administered subcutaneously once daily for the first 30 days with a maximum daily dose of 18,000 IU, followed by 150 IU/kg. The standard dosing schedules of anticoagulation were determined by the latest version of NCCN and ASCO guidelines based on pivotal trials regarding CA-VTE [14]. The dosages could be temporarily withheld or reduced in case of a platelet count lower than 75,000/μL, clinical evidence of bleeding, or any condition associated with an increased risk of bleeding according to the protocol. In all patients, DOAC or dalteparin treatment was to be continued for 6 months and then discontinued unless VTE persisted or recurred.

### 2.3. Outcomes

The primary safety outcome was CRB incidence during the 6-month study. Secondary safety outcomes included incidence of major bleeding and total bleeding. CRB was classified into MB and clinically relevant nonmajor bleeding, as reported previously [11,15,16] (Table S1). MB was defined as acute and clinically overt bleeding accompanied by one or more of the following: contribution to death, occurrence in critical sites such as intracranial or retroperitoneal sites, a decrease of ≥2 g/dL in hemoglobin levels, or the need for transfusion of ≥2 units of red blood cells. If the criteria for MB were not fulfilled, clinically relevant nonmajor bleeding was defined as acute and clinically overt bleeding with one or more of the following: the need for medical intervention, unscheduled contact with a physician, interruption or discontinuation of anticoagulation, or impairment of activities of daily life. Minor bleeding was defined as overt bleeding not meeting the criteria for CRB. Total bleeding was defined as all reported bleeding, regardless of severity. Adverse events experienced from study entry until 30 days after the end of the study were recorded according to the National Cancer Institute Common Terminology Criteria for Adverse Events (version 4.03).

The secondary safety outcomes were time to bleeding (MB, CRB, total bleeding), and efficacy outcomes were the rate and time to symptomatic or incidental thromboembolic recurrence, including DVT or PTE in the lower or upper extremities (jugular, innominate, subclavian, axillary, brachial). These were objectively confirmed on CT, ultrasonography, or perfusion scan. An arterial thromboembolism during anticoagulation, such as myocardial or cerebral infarction or peripheral arterial embolism, was also classified as a secondary efficacy event.

Although the study protocol did not specify central adjudication of the study outcomes, an investigator-reported major bleeding and clinically relevant bleeding were subsequently adjudicated by a central committee before the interim analysis, although it was not blinded for treatment allocation.

### 2.4. Surveillance and Follow-Up

Patients were scheduled to visit clinics at enrolment and every 2–4 weeks during anticoagulation treatment, which was planned because the study population consisted of advanced or metastatic cancer patients subject for chemotherapy usually requiring clinic visit every 1–4 weeks, and then followed up every 3 months for 1 year or until death. Additional visits were temporarily planned if bleeding events or new symptoms of VTE occurred or if the investigator determined that evaluation was needed. Clinical examination and laboratory testing, including complete blood count, chemistry, and prothrombin time/activated partial thromboplastin time, were performed every visit, and imaging tests including CT, ultrasonography, or lung perfusion scan were performed at study completion or any time when patients had symptoms suggesting recurrent/aggravated CA-VTE. The regular CT scans were performed 6–8 weeks for disease evaluation as a part of routine practice in patients with chemotherapy, and at least every 3 months in patients without chemotherapy.

### 2.5. Statistical Analysis

We hypothesized that the rate of CRB events would be higher in the DOAC group than in the dalteparin group, with an expected difference of 15% based on our previous study [15]. We planned to enroll 164 patients (82 to each arm), assuming CRB incidence of 14% in the dalteparin group and 29% in the DOAC group during the 6-month anticoagulation period (with 80% power at a one-sided type I error rate of 0.1 and 5% dropout rate). One prespecified interim analysis was planned for superiority of dalteparin over DOAC in terms of safety, with a prespecified significant, one-sided *p* value (<0.020) for the date at which half of the expected CRB (*n* = 19/37) occurred. At the time of database lock for interim analysis, 21 patients developed CRB (56.7% expected CRB) among 92 patients enrolled; the boundary for declaring superiority for CRB (i.e., lower CRB) was adjusted to a one-sided *p* value < 0.027, based on the O’Brien–Fleming type alpha spending function [17].

Interim analysis was conducted in the intention-to-treat (ITT) set, which was defined as all patients who met the inclusion/exclusion criteria and were randomly assigned. The results of interim analysis were reviewed by an independent data monitoring committee (IDMC), and the trial was to be stopped early if dalteparin showed superior safety to DOAC in the primary outcome. An independent statistician prepared and presented summary data for the interim analysis to IDMC, which was composed of three independent medical oncologists and one independent statistician. The final analysis was performed in the FAS, defined as patients with at least one dose of the assigned treatment after randomization and meeting inclusion/exclusion criteria, when the last enrolled patient was followed up for at least 6 months. The robustness of the results was confirmed in the ITT set. Four patients switched anticoagulants before any total event at the patients’ preferences; as such, the dataset according to the administered drug during bleeding events was also analyzed, and prespecified subgroup analyses were performed based on age, sex, concurrent active chemotherapy, and presence of gastrointestinal mucosal invasion. The cancer involvement at the GI mucosa indicated primary cancer lesion itself, cancer invasion on adjacent GI mucosa, or metastatic mucosal lesion from non-GI tract cancer, which were determined by the endoscopy.

The chi-square test or Fisher’s exact test was used for categorical, and Mann–Whitney U-test for quantitative data, respectively. The time to event was calculated from the date of randomization to the dates of the first bleeding episode and recurrent CA-VTE. Patients without any event were censored at the time of the last follow-up. The Kaplan–Meier curves were used to estimate the time to event and compared using the log-rank test. Cox proportional hazards models were used to estimate the correlation between the anticoagulation type and the time to event. Multivariate analysis included age, sex, and significant factors (*p* < 0.1) in the univariate analysis. A one-sided *p* value < 0.027 was considered significant for the evaluation of CRB rates as the primary endpoint between groups, and a two-sided *p* value < 0.05 was considered significant for the other all statistical analysis.

## 3. Results

### 3.1. Patient Characteristics

**Figure 1:**
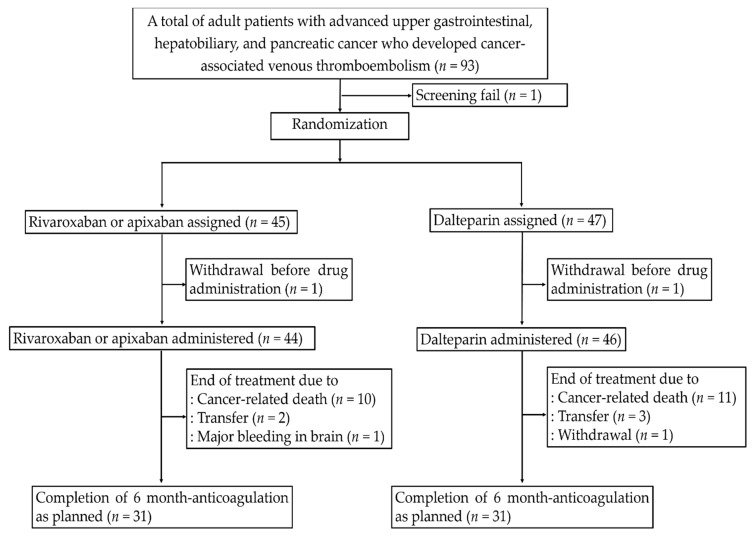
Consort diagram.

Between August 2017 and June 2020, a total of 93 patients were screened from five sites in Korea. One patient who failed the screening test was excluded, and 92 patients were randomly assigned to either the DOAC (*n* = 45) or dalteparin group (*n* = 47) (Figure 1). One patient in each group withdrew from the trial before drug administration. Consequently, 90 patients (44 in the DOAC group and 46 in the dalteparin group) were included in the final safety and efficacy analysis. The database was locked on December 2020, and final analysis was performed. The baseline characteristics of the patients in FAS were reasonably comparable between groups (Table 1). There were symptomatic CA-VTE (*n* = 46, 51.1%) and incidental CA-VTE (*n* = 44, 48.9%). Among 23 patients with DVT, most of the patients had lower extremities DVT except for only two right internal jugular DVT in the dalteparin group. Most patients had metastatic disease (78.9%) and received palliative chemotherapy during anticoagulation (90.0%). The median duration of the assigned treatment was 146 days (interquartile range, 42–183 days) in the DOAC group and 155 days (interquartile range, 62–183 days) in the dalteparin group (*p* = 0.583). Sixty-two patients (67.4%) completed 6 months of treatment. The main reason for not completing the treatment in both arms was cancer-related death (Figure 1).

### 3.2. Primary Safety Outcomes

The interim analysis for the primary safety outcome was conducted in August 2020 for the 92 enrolled patients based on data as of June 2020 (median follow-up, 13.0 months). The IDMC recommended early termination of the study according to the prespecified stopping criteria on the basis of superiority, with 21 (53.8%) of the expected 39 CRB events reported. The primary outcome of CRB occurred in 15 of 44 patients (34.1%) in the DOAC group and in 6 of 46 patients (13.0%) in the dalteparin group (*p* = 0.018). MB occurred in eight patients (18.2%) in the DOAC group and in two patients (4.3%) in the dalteparin group (*p* = 0.047) (Table 2). The most common CRB site was the GI tract (52.4%, 6/21) followed by the GU tract (28.6%, 6/21) (GI tract (60.0%, 9/15 DOAC vs. 33.3%, 2/6 dalteparin) and GU tract (20.0%, 3/15 DOAC vs. 50.0%, 3/6 dalteparin)) (Table 3). Among 10 patients with MB (8, DOAC group; 2, dalteparin group), GI tract bleeding occurred in six patients (75%) in the DOAC group and in two (100%) in the dalteparin group (Table 3). There was one episode of intracranial hemorrhage that occurred approximately 3 months after initiation of rivaroxaban.

In the subgroup analysis regarding the comparison between each rivaroxaban, apixaban, and dalteparin group, both rivaroxaban and apixaban increased risk of MB and CRB compared to dalteparin (MB, 16.1% for rivaroxaban and 23.1% for apixaban vs. 4.3% for dalteparin; *p* = 0.078 for rivaroxaban and *p* = 0.066 for apixaban compared to dalteparin) (CRB, 29.0% and 46.2% vs. 13.0%, respectively; *p* = 0.082 for rivaroxaban and *p* = 0.009 for apixaban compared to dalteparin) (Table S2). In terms of the time to MB or CRB, rivaroxaban showed a trend of being a risk factor for MB (HR 3.89; *p* = 0.104) and CRB (HR 2.37; *p* = 0.102) compared to dalteparin (Table S2). Apixaban showed a trend of being a risk factor for MB (HR 5.28; *p* = 0.068) and was a significant factor for CRB (HR 3.93; *p* = 0.018) (Table S2). After multivariate analysis with cancer involvement at GI mucosa, apixaban showed a trend of significant risk factor for CRB compared to dalteparin (HR 3.12; *p* = 0.055). There were no significant differences in MB (16.1% vs. 23.1%; *p* = 0.676) and CRB (29.0% vs. 46.2%; *p* = 0.313) between rivaroxaban and apixaban (Table S2). In terms of the time to MB or CRB events, there were also no significant differences between rivaroxaban and apixaban (HR (of apixaban compared to rivaroxaban) for MB 1.31; *p* = 0.710 and HR for CRB 1.58; *p* = 0.386) (Table S2).

### 3.3. Secondary Safety and Efficacy Outcomes

**Figure 2:**
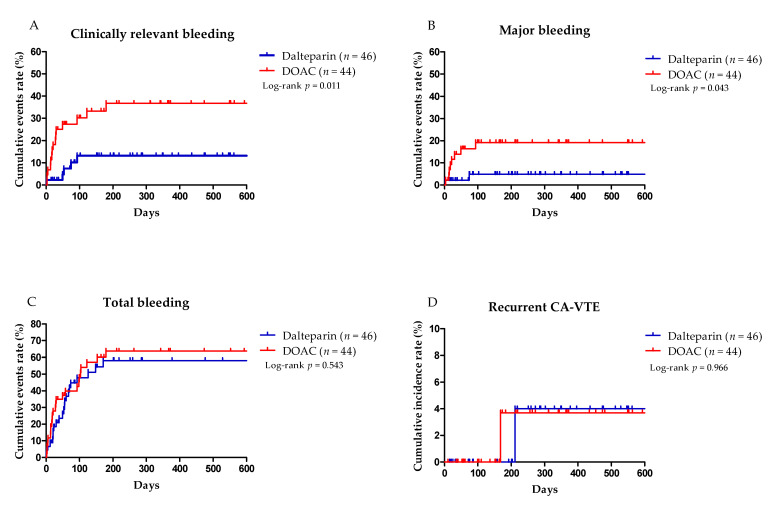
The Kaplan–Meier curves of the time to (**A**) clinically relevant bleeding (major and nonmajor bleeding), (**B**) major bleeding, (**C**) total events of bleeding, and (**D**) recurrent cancer-associated venous thromboembolism in full analysis set.

The time to CRB was significantly longer in the DOAC group than in the dalteparin group (log-rank test *p* = 0.011 and HR 2.83, 95% confidence interval (CI) 1.10–7.30; *p* = 0.031) (Figure 2A and Table 2). After multivariate analysis with age, sex, and cancer involvement at GI mucosa, DOAC therapy remained a significant risk factor for CRB (adjusted HR 2.83, 95% CI 1.09–7.29; *p* = 0.031) (Table 2). The time to MB was longer in the DOAC group than in the dalteparin group (log-rank test *p* = 0.043 and HR 4.32, 95% CI 0.92–20.36; *p* = 0.064) (Figure 2B and Table 2). After multivariate analysis with age, sex, and cancer involvement at GI mucosa, DOAC therapy showed a trend of being a risk factor for MB (adjusted HR 4.05, 95% CI 0.86–19.11; *p* = 0.077) (Table 2). Clinically relevant nonmajor bleeding occurred in eight patients (18.2%) in the DOAC group and four patients (8.7%) in the dalteparin group (*p* = 0.186) (HR 2.11, 95% CI 0.64–7.02; *p* = 0.222) (Table 2). There were no differences in total bleeding events between the DOAC and dalteparin groups (59.1% vs. 50.0%; *p* = 0.387) (Table 2), and the time to total bleeding events was not significantly different between groups (HR 1.19, 95% CI 0.68–2.09; *p* = 0.545) (Table 2 and Figure 2C). Table 4 summarizes the univariate analysis of clinicolaboratory factors for MB and CRB. Cancer involvement at GI mucosa (HR 2.57, 95% CI 1.06–6.21; *p* = 0.036) was significantly associated with risk of CRB, but it did not remain a significant factor in multivariate analysis (HR 2.08, 95% CI 0.84–5.15; *p* = 0.115). Among 38 patients with cancer involvement at GI mucosa, stomach (*n* = 21) was the most common site, followed by esophagus (*n* = 7), duodenum (*n* = 7), ampulla of vater (*n* = 2), and colon (*n* = 1) (Table S3). The characteristics of endoscopic findings and intervention was summarized in Table S3. No other risk factors for MB and CRB were observed.

Recurrent CA-VTE occurred in one patient (2.3%) in the DOAC group and one patient (2.2%) in the dalteparin group (*p* = 1.000) (Table 2), and the time to recurrent CA-VTE was not significantly different between groups (HR 1.06, 95% CI 0.07–16.98; *p* = 0.966) (Table 2 and Figure 2D).

In the ITT set, the baseline characteristics of the patients were well balanced between groups (Table S4). CRB and MB occurred in 15 patients (33.3%) and 8 patients (17.8%) in the DOAC group (*p* = 0.019) and 6 patients (12.8%) and 2 patients (4.3%) in the dalteparin group (*p* = 0.048), respectively (Table S5). Time to CRB or MB was significantly longer in the DOAC group than in the dalteparin group (*p* = 0.011 and *p* = 0.043) (Figure S1). The time to recurrent CA-VTE was not significantly different between groups (*p* = 0.966) (Figure S1). After multivariate analysis with age, sex, and cancer involvement at GI mucosa, DOAC therapy remained a significant risk factor for CRB (adjusted HR 2.83, 95% CI 1.09–7.29; *p* = 0.031) and showed a trend of being a risk factor for MB (adjusted HR 4.05, 95% CI 0.86–19.11; *p* = 0.077) (Table S5). Cancer involvement at GI mucosa (HR 2.57, 95% CI 1.06–6.20; *p* = 0.036) was also significantly associated with risk of CRB (Table S6).

### 3.4. Subgroup Analysis Based on the Drug Administered during Bleeding Events

Four patients switched anticoagulant during early study periods due to their clinical situation, and two of them developed CRB during modified drug administration. Among four patients, three patients switched from dalteparin to DOAC due to patient preference for oral convenience, and CRB occurred in two patients; one patient developed GI bleeding at 74 days during rivaroxaban administration after only four days of dalteparin; the other patient developed hematuria at four days during apixaban administration after only one dose of dalteparin. Among four patients, the last patient switched to dalteparin after 1 week of DOAC therapy due to poor oral intake after septic shock, and no CRB occurred.

**Figure 3:**
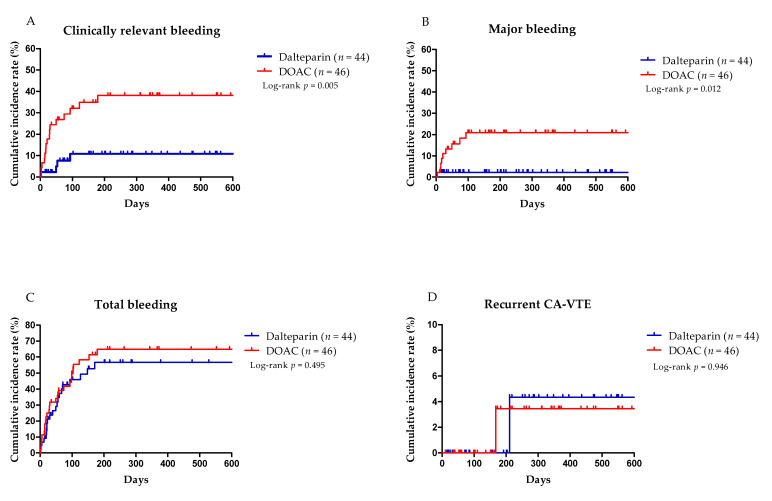
The Kaplan–Meier curves of the time to (**A**) clinically relevant bleeding (major and nonmajor bleeding), (**B**) major bleeding, (**C**) total events of bleeding, and (**D**) recurrent cancer-associated venous thromboembolism according to the administered drug during bleeding events in full analysis set.

Table 5 summarizes the rates of bleeding events and recurrent CA-VTE according to the administered drugs. The primary outcome of CRB occurred in 17/46 patients (37.0%) in the DOAC group and in 4/44 patients (9.1%) in the dalteparin group (*p* = 0.002). MB occurred in nine patients (19.6%) in the DOAC group and in one patient (2.3%) in the dalteparin group (*p* = 0.015) (Table 5). The time to CRB and MB was significantly longer in the DOAC group than in the dalteparin group (*p* = 0.005 and *p* = 0.012, respectively) and the time to recurrent CA-VTE was not significantly different between groups (*p* = 0.946) (Figure 3). After multivariate analysis with age, sex, and cancer involvement at GI mucosa, the time to CRB (adjusted HR 4.51, 95% CI 1.52–13.44; *p* = 0.007) and MB (adjusted HR 8.88, 95% CI 1.12–70.17; *p* = 0.038) was significantly longer in the DOAC group than in the dalteparin group (Table 5).

## 4. Discussion

The primary endpoint of this trial was met where the rate of CRB was significantly higher in the rivaroxaban/apixaban group than in the dalteparin group in active advanced upper GI tract, hepatobiliary, and pancreatic cancer (34.1% vs. 13.0%; *p* = 0.018). The time to CRB was significantly longer in the rivaroxaban/apixaban group than in the dalteparin group (adjusted HR 2.83, 95% CI 1.09–7.29; *p* = 0.031). MB also occurred more often in the rivaroxaban/apixaban group than in the dalteparin group (18.2% vs. 4.3%; *p* = 0.047). All these findings were confirmed in the ITT set. According to the administered drug during bleeding events, the significant differences in the rates of both events became more prominent (CRB: 37.0% vs. 9.1%; *p* = 0.002 and MB: 19.6% vs. 2.3%; *p* = 0.015), and HRs for time to bleeding increased by 1.5–2-fold (adjusted HR for CRB 4.51; *p* = 0.007 and adjusted HR for MB 8.88; *p* = 0.038). There was no difference in CA-VTE recurrence between the two groups (2.3%, DOAC group; 2.2%, dalteparin group; *p* = 1.000).

These findings confirm the results of our previous retrospective study, which compared rivaroxaban with LMWH in the same study population; the incidence of CRB was 31.9% for rivaroxaban and 14.3% for LMWH (two-sided *p* = 0.006), and the incidence of MB was 17.4% and 7.6% (two-sided *p* = 0.048), respectively. Meanwhile, recurrent or aggravated VTE occurred at similar rates in both groups (2.9% in rivaroxaban vs. 1.0% in LMWH; *p* = 0.563) [15]. Although recent cancer-specific clinical trials comparing DOAC and LMWH have shown DOAC has noninferior or superior efficacy to dalteparin for CA-VTE, there were some conflicting results regarding bleeding risk [10,11,12,13]. The SELECT-D study showed rivaroxaban was associated with relatively low VTE recurrence compared with dalteparin (6-month cumulative rate, 4% vs. 11%; HR = 0.43, 95% CI 0.19–0.99) but had higher CRNMB (6-month cumulative rate, 13% vs. 4%; HR = 3.76, 95% CI 1.63–8.69) and similar MB (6-month cumulative rate, 6% vs. 4%; HR = 1.83, 95% CI 0.68–4.96) [11]. The Caravaggio study showed that apixaban was noninferior to dalteparin for recurrent CA-VTE (HR = 0.63; 95% CI 0.37–1.07; *p* < 0.001 for noninferiority; *p* = 0.09 for superiority) without an increased risk of MB (3.8% in apixaban vs. 4.0% in dalteparin; HR = 0.82, 95% CI 0.40–1.69, *p* = 0.60) or CRNMB (9.0% in apixaban vs. 6.0% in dalteparin; HR = 1.42, 95% CI 0.88–2.30) [12]. The Hokusai VTE Cancer study showed that edoxaban was noninferior to dalteparin with respect to the composite outcome of recurrent VTE or MB (HR = 0.97, 95% CI 0.70–1.36; *p* = 0.006 for noninferiority; *p* = 0.87 for superiority), but the rate of MB was higher for edoxaban than dalteparin (6.9% vs. 4.0%; HR = 1.77, 95% CI 1.03–3.04; *p* = 0.04) [10]. The ADAM VTE trial, which hypothesized that apixaban was associated with a significantly lower MB rate compared to dalteparin, failed to show the superiority of apixaban in terms of MB (0% in apixaban vs. 1.4% in dalteparin; *p* = 0.138) or CRB (6.2% in apixaban vs. 6.3% in dalteparin, *p* = 0.881). Instead, CRNMB was numerically higher for apixaban (6.2%) than dalteparin (4.9%), while VTE recurrence was lower for apixaban (0.7%) than for dalteparin (6.3%) (HR = 0.10, *p* = 0.028) [13].

These conflicting results of bleeding risk might be partly associated with the heterogenous cancer types and cancer status included in the trials. Most studies included patients with a history of cancer diagnosed within 2 years before enrolment, and the definition of active cancer, including cancer diagnosis and treatment in the previous 6 months, was used. This suggests that patients without a currently existing cancer lesion at the beginning or during anticoagulation could also have been enrolled in those trials, which might have influenced bleeding risk. GI cancer, especially upper GI tract or pancreatobiliary cancers, is well known for having the highest VTE risk and the highest bleeding risk, regardless of anticoagulation [4,5]. In the Hokusai VTE cancer study, patients with GI cancer had a higher risk of MB during edoxaban treatment than dalteparin treatment (13.2% with edoxaban vs. 2.4% with dalteparin in GI cancer; 4.7% with edoxaban vs. 4.5% with dalteparin in non-GI cancer (interaction *p* = 0.02)) [10]. Most major GI bleeding in the edoxaban group occurred at upper GI sites (85.0%). Likewise, in the SELECT-D study, most MB events were in the upper GI tract, including the esophagus or stomach. Interim safety analysis showed an increased trend in MB toward rivaroxaban in the 19 patients with esophagus or gastroesophageal junction cancer (36% in rivaroxaban vs. 5% in dalteparin), leading to subsequent exclusion of these cancer types from enrolment as a precautionary measure. In the current PRIORITY study focusing on upper GI tract and hepatopancreatobiliary cancers with currently existing advanced or metastatic cancer lesions, MB (18.2%) and CRNMB (18.2%) seemed to occur more often in the DOAC group compared with in previous cancer-specific trials, in which the most common cancers were colorectal and lung cancers (0–6.9% for MB and 6.2–14.6% for CRNMB); meanwhile, this result was not as obvious for dalteparin (MB, 4.3% for dalteparin in the PRIORITY study vs. 1.4–4.0%; CRNMB, 8.7% for dalteparin vs. 3.4–11.1%). These results suggest that these cancer types might be susceptible to bleeding with DOAC. Notably, in the PRIORITY study, more than half (52.4%) of CRB and 80% of MB events occurred in the GI tract. Cancer involvement at GI mucosa was a significant risk factor for CRB (HR = 2.57; *p* = 0.036) rather than the cancer type itself, which may be contributed to by absence of cancer involvement at GI mucosa after prior surgery of primary sites such as gastrectomy. Moreover, the GI mucosa damaged by chemotherapy targeting GI tract cancer, together with high concentrations of DOAC in the GI lumen, may be responsible for bleeding events [18,19]. These findings could help in guiding optimal anticoagulation therapy for CA-VTE, particularly in patients at high risk for bleeding because of the cancer site.

Interestingly, in the Caravaggio trial [12], apixaban did not significantly increase the risk of bleeding events compared with dalteparin in terms of MB (3.8% vs. 4.0%), CRB (12.2% vs. 9.7%), and clinically relevant nonmajor bleeding (9.0% vs. 6.0%). The ADAM-VTE randomized trial also reported that MB occurred in 0% of 145 patients receiving apixaban compared with 1.4% of 142 patients receiving dalteparin (*p* = 0.138), and CRB rates were 6% for both groups [13]. These results suggest that apixaban, unlike other DOACs [10,11,12,13], appears to have a similar risk of bleeding in CA-VTE treatment compared with dalteparin. However, in our study, apixaban increased risk of MB and CRB compared to dalteparin (MB, 23.1% vs. 4.3%; *p* = 0.066 and CRB, 46.2% vs. 13.0%; *p* = 0.009). In terms of the time to MB or CRB, apixaban showed a trend of being a risk factor for MB (HR 5.28; *p* = 0.068) and was a significant risk factor for CRB (HR 3.93; *p* = 0.018). After multivariate analysis with cancer involvement at GI mucosa, apixaban showed a trend of significant risk factor for CRB compared to dalteparin (HR 3.12; *p* = 0.055). Recently, a study using prospectively collected data in 1392 CA-VTE patients at the Mayo clinic reported that apixaban had a higher rate of MB in GI tract cancer compared to non-GI tract cancer patients and compared to enoxaparin (other LMWH) in GI tract cancer patients, whereas rivaroxaban showed no increased risk of MB in GI tract cancer compared to non-GI tract cancer patients [20]. Furthermore, the Caravaggio and ADAM-VTE trials included fewer patients with upper GI tract cancer compared to the Hokusai VTE cancer and SELECT-D trials. The use of apixaban still requires caution, and further studies regarding which DOAC is likely to have a lower risk of bleeding are necessary, in particular, in patients with higher risks of bleeding.

On the contrary to the MB or CRB, there were no significant differences in total bleeding between the DOAC and dalteparin groups (59.1% vs. 50.0%; *p* = 0.387). However, the bruise on the injection area in the dalteparin group was also counted in total bleeding, which might have unfavorably contributed to the dalteparin group, resulting from the differences in administration form. With the exception of these events in the dalteparin group, total bleeding also occurred significantly more often in the DOAC group than in the dalteparin group (59.1% vs. 34.8%; *p* = 0.034). The rates of recurrence seemed to be lower than the previous pivotal trials (Hokusai VTE cancer, SELECT-D, Caravaggio, and ADAM-VTE). In our study, recurrent CA-VTE, which was the newly developed CA-VTE, was only counted as the efficacy outcome. However, the previous pivotal trials included not only recurrent CA-VTE but also aggravation of pre-existing CA-VTE. After including aggravation of CA-VTE, recurrent or aggravation of CA-VTE occurred in two patients (4.5%) in the DOAC group and four patients (8.7%) in the dalteparin group (*p* = 0.677), in line with the previous trials. These may be also explained by the different ethnic/racial groups and active cancer status. It is known that Asian patients have a significantly lower cancer-associated VTE incidence than Caucasian patients [21,22]. A study in 1732 Japanese patients with VTE reported that VTE recurrence was 1.8% with edoxaban [23]. Furthermore, as this study focused on patients with currently active and metastatic cancers, the duration of follow-up could be relatively short due to clinical deterioration with disease progression and death, which could come first before recurrent CA-VTE.

This study has some limitations. First, despite a multicenter study, most patient enrollment was concentrated at one center, the Asan Medical Center, which has the largest volume of cancer patients in Korea, and the enrollment duration was relatively long due to frequent patient refusal, from patient preference for oral anticoagulation and stringent eligibility criteria of high-risk cancer types with currently active and advanced cancers. Second, as an open-label study design, as it is inevitable to compare the oral rivaroxaban/apixaban with subcutaneous dalteparin, a central adjudication committee nonblindedly confirmed the outcomes with intrinsic limitations. Third, there were numerical differences in cancer types (GI tract vs. hepatobiliary pancreas cancer) of baseline characteristics between the DOAC and dalteparin groups, despite statistical nonsignificance. Fourth, rivaroxaban or apixaban was determined at the physician’s discretion and edoxaban use was not included to reduce variations regarding treatment options in the DOAC group. Lastly, the sample size is relatively small compared with the previous pivotal trials since early termination of the study after interim analysis, meeting statistically prespecified stopping criteria on the safety issue. This interim analysis was necessary for ethical issues and approved by each IRB of participating centers. However, this study may be valuable to focus on high-risk cancer types and currently active advanced cancers, and to evaluate the safety issue as primary endpoint.

## 5. Conclusion

The current study suggests that DOAC therapy further increased the risk of bleeding compared with dalteparin in patients with active advanced upper GI tract, hepatobiliary, or pancreatic cancer. Thereby, significant caution is necessary when selecting anticoagulation treatment for CA-VTE in high-risk patients.

## Figures and Tables

**Table 1:** Baseline characteristics in full analysis set.

	DOAC (*n* = 44)	Dalteparin (*n* = 46)	*p*
Median age, years (range)	64 (39−77)	63 (42−78)	0.864
Sex			0.662
Male	25 (56.8)	23 (50.0)	
Female	19 (43.2)	23 (50.0)	
BMI	22.5 ± 3.3	22.4 ± 3.1	0.916
CA-VTE			0.250
Deep vein thromboembolism	4 (9.1)	10 (21.7)	
Pulmonary thromboembolism	35 (79.5)	32 (69.6)	
Both	5 (11.4)	4 (8.7)	
Tumor types			0.453
Esophageal cancer	8 (18.2)	5 (10.9)	
Gastric cancer	19 (43.2)	18 (39.1)	
Ampulla of vater cancer	1 (2.3)	1 (2.2)	
Duodenal cancer	0 (0)	1 (2.2)	
Hepatocellular carcinoma	2 (4.5)	0 (0)	
Biliary cancer	8 (18.2)	9 (19.6)	
Pancreatic cancer	6 (13.6)	12 (26.1)	
ECOG PS			0.662
0–1	37 (84.1)	36 (78.3)	
≥2	7 (15.9)	10 (21.7)	
Metastatic disease	38 (86.4)	33 (71.7)	0.122
Chemotherapy during anticoagulation	41 (93.2)	40 (87.0)	0.486
Lines of chemotherapy during anticoagulation *			0.447
First-line	24 (58.5)	28 (70.0)	
Second-line	12 (29.3)	7 (17.5)	
Third or later line	5 (12.2)	5 (12.5)	
Radiotherapy during anticoagulation	2 (4.5)	1 (2.2)	0.969

Abbreviation: DOAC, direct oral anticoagulant; BMI, body mass index; CA-VTE, cancer-associated venous thromboembolism; GI, gastrointestinal; ECOG PS, Eastern Cooperative Oncology Group Performance Status. * Percentages were calculated from 81 patients treated with chemotherapy during anticoagulation.

**Table 2:** Recurrent cancer-associated venous thromboembolism and bleeding events in the full analysis set.

	DOAC (*n* = 44, %)	Dalteparin (*n* = 46, %)	p *	HR (95% CI)	p **	Adjusted HR *** (95% CI)	p **
Recurrent CA-VTE	1 (2.3)	1 (2.2)	1.000	1.06 (0.07–16.98)	0.966	0.97 (0.05–19.23)	0.985
Category of bleeding events							
Major bleeding	8 (18.2)	2 (4.3)	0.047	4.32 (0.92–20.36)	0.064	4.05 (0.86–19.11)	0.077
Clinically relevant nonmajor bleeding	8 (18.2)	4 (8.7)	0.186	2.11 (0.64–7.02)	0.222	1.70 (0.49–5.88)	0.404
Clinically relevant bleeding	15 (34.1)	6 (13.0)	0.017	2.83 (1.10–7.30)	0.031	2.83 (1.09–7.29)	0.031
Total bleeding	26 (59.1)	23 (50.0)	0.387	1.19 (0.68–2.09)	0.545	1.12 (0.61–2.04)	0.719

Abbreviation: CA-VTE, cancer-associated venous thromboembolism; HR, hazard ratio; CI, confidence interval. * Two-sided *p* values were estimated using the chi-square test or Fisher’s exact test. A one-sided *p* value was estimated using the chi-squared test or Fisher’s exact test. ** Two-sided *p* values were estimated using the Cox proportional regression model. *** Hazard ratio was adjusted with age, sex, and cancer involvement at gastrointestinal mucosa.

**Table 3:** Bleeding sites according to type of anticoagulation in patients with major bleeding or clinically relevant bleeding in the full-analysis set.

	All (*n* = 90, %)	DOAC (*n* = 44, %)	Dalteparin (*n* = 46, %)	*p* *
Major bleeding	10/90 (11.1)	8/44 (18.2)	2/46 (4.3)	0.047
				0.732
GI tract	8/10 (80.0)	6/8 (75.0)	2/2 (100.0)	
Brain	1/10 (10.0)	1/8 (12.5)	0 (0)	
Vagina	1/10 (10.0)	1/8 (12.5)	0 (0)	
Clinically relevant bleeding		15/44 (34.1)	6/46 (13.0)	0.018
				0.528
GI tract	11/21 (52.4)	9/15 (60.0)	2/6 (33.3)	
GU tract	6/21 (28.6)	3/15 (20.0)	3/6 (50.0)	
Brain	1/21 (4.8)	1/15 (6.7)	0/6 (0)	
Vagina	2/21 (9.5)	1/15 (6.7)	1/6 (16.7)	
Epistaxis	1/21 (4.8)	1/15 (6.7)	0/6 (0)	

Abbreviation: DOAC, direct oral anticoagulant; GI, gastrointestinal; GU, genitourinary. * *p* values were estimated using the chi-square test or Fisher’s exact test.

**Table 4:** Univariate analysis for major bleeding and clinically relevant bleeding in the full analysis set.

	Major Bleeding		Clinically Relevant Bleeding	
	HR (95% CI)	p *	HR (95% CI)	p *
Male vs. Female	2.01 (0.52–7.79)	0.311	1.80 (0.73–4.47)	0.203
Age ≥65 years vs. <65 years	0.28 (0.06–1.30)	0.103	0.55 (0.22–1.36)	0.197
ECOG PS ≥2 vs. ECOG PS 0–1	0.56 (0.07–4.44)	0.584	1.30 (0.44–3.89)	0.638
BMI (<18.5) vs. BMI (≥18.5)	0.90 (0.11–7.20)	0.921	1.33 (0.39–4.53)	0.651
Primary cancer type		0.397		0.246
Upper GI tract cancer	1		1	
Hepatobiliary and pancreas cancer	1.71 (0.49–5.94)		1.66 (0.70–3.94)	
Hemoglobin <9.0 g/dL vs. ≥9.0 g/dL	2.21 (0.57–8.55)	0.252	1.66 (0.61–4.55)	0.322
Platelets <100 × 10^6^/µL vs. ≥100 × 10^6^/µL	2.37 (0.50–11.17)	0.275	0.94 (0.22–4.02)	0.928
Cr clearance <60 mL/min vs. ≥60 mL/min	1.10 (0.23–5.27)	0.910	1.04 (0.35–3.12)	0.945
Albumin <3.5 g/dL vs. ≥3.5 g/dL	1.87 (0.48–7.25)	0.366	1.68 (0.68–4.19)	0.262
Activated partial thromboplastin time (aPTT) >35 s vs. ≤35 s	1.14 (0.14–8.99)	0.901	0.51 (0.07–3.83)	0.515
Anticancer systemic therapy during anticoagulation (yes vs. no)	23.04 (0.0–798,399.18)	0.556	22.96 (0.01–38,485.883)	0.408
Treatment lines of systemic therapy during anticoagulation				
First-line	1		1	
Second line	1.64 (0.39–6.86)	0.499	2.13 (0.81–5.61)	0.127
Third or later line	2.45 (0.47–12.68)	0.286	2.58 (0.80–8.30)	0.111
Radiotherapy during anticoagulation (yes vs. no)	4.07 (0.51–32.17)	0.184	1.65 (0.22–12.35)	0.624
Cancer involvement at GI mucosa (yes vs. no)	2.27 (0.64–8.06)	0.204	2.57 (1.06–6.21)	0.036
Type of anticoagulant (DOAC vs. dalteparin)	4.32 (0.92–20.36)	0.064	2.83 (1.10–7.30)	0.031

Abbreviations: HR, hazard ratio; CI, confidence interval; ECOG PS, Eastern Cooperative Oncology Group Performance Status; BMI, body mass index; Cr, creatinine; GI, gastrointestinal; DOAC, direct oral anticoagulant. * Two-sided *p* values were estimated using the Cox proportional hazards model.

**Table 5:** Recurrent cancer-associated venous thromboembolism and bleeding events according to administered drugs during bleeding events in the full analysis set.

	DOAC (*n* = 46 †, %)	Dalteparin (*n* = 44 †, %)	p *	HR (95% CI)	p **	Adjusted HR *** (95% CI)	p **
Recurrent CA-VTE	1 (2.2)	1 (2.2)	1.000	0.91 (0.06–14.52)	0.946	0.87 (0.05–16.34)	0.924
Category of bleeding events							
Major bleeding	9 (19.6)	1 (2.3)	0.015	8.89 (1.13–70.17)	0.038	8.88 (1.12–70.17)	0.038
Clinically relevant nonmajor bleeding	9 (19.6)	3 (6.8)	0.120	2.93 (0.80–10.85)	0.106	2.42 (0.64–9.22)	0.195
Clinically relevant bleeding	17 (37.0)	4 (9.1)	0.002	4.52 (1.52–13.44)	0.007	4.51 (1.52–13.44)	0.007
Total bleeding	28 (60.9)	21 (47.7)	0.211	1.31 (0.75–2.31)	0.346	1.21 (0.67–2.19)	0.525

Abbreviations: CA-VTE, cancer-associated venous thromboembolism; HR, hazard ratio; CI, confidence interval. † Four patients initially switched anticoagulant depending on clinical situation at the physician’s discretion; dalteparin → DOAC (*n* = 3) and DOAC → dalteparin (*n* = 1). * *p* values were estimated using the chi-squared test or Fisher’s exact test. A one-sided *p* value was estimated using the chi-squared test or Fisher’s exact test. ** *p* values were estimated using the Cox proportional regression model. *** Hazard ratio was adjusted with age, sex, and cancer involvement at gastrointestinal mucosa.

## Data Availability

The datasets generated during and/or analyzed during the current study are available from the corresponding author (SRP) on reasonable request. The data are not publicly available due to them containing information that could compromise research participant privacy/consent.

## Supplementary Materials

The following supporting information can be downloaded at: https://www.mdpi.com/article/10.3390/cancers14030559/s1. Figure S1: The Kaplan–Meier curves of the time to (A) clinically relevant bleeding (major and non-major bleeding), (B) major bleeding, (C) total bleeding events, and (D) recurrent cancer-associated venous thromboembolism in the intent-to-treat set.; Table S1: Definition of recurrence of venous thromboembolism and bleeding; Table S2: Recurrent cancer-associated venous thromboembolism and bleeding events between the rivaroxaban, apixaban, and dalteparin groups; Table S3: Characteristics of cancer involvement at gastrointestinal mucosa; Table S4: Baseline characteristics in the intent-to-treat set; Table S5: Recurrent cancer-associated venous thromboembolism and bleeding events in intent-to-treat set; Table S6: Univariate analysis for major bleeding and clinically relevant bleeding in intent-to-treat set.

## References

[1] Baron J.A., Gridley G., Weiderpass E., Nyrén O., Linet M. (1998). Venous thromboembolism and cancer. Lancet.

[2] Prandoni P., Lensing A.W., Piccioli A., Bernardi E., Simioni P., Girolami B., Marchiori A., Sabbion P., Prins M.H., Noventa F. (2002). Recurrent venous thromboembolism and bleeding complications during anticoagulant treatment in patients with cancer and venous thrombosis. Blood.

[3] Hutten B.A., Prins M.H., Gent M., Ginsberg J., Tijssen J.G., Büller H.R. (2000). Incidence of recurrent thromboembolic and bleeding complications among patients with venous thromboembolism in relation to both malignancy and achieved international normalized ratio: A retrospective analysis. J. Clin. Oncol. Off. J. Am. Soc. Clin. Oncol..

[4] Khorana A.A., Kuderer N.M., Culakova E., Lyman G.H., Francis C.W. (2008). Development and validation of a predictive model for chemotherapy-associated thrombosis. Blood.

[5] Patell R., Gutierrez A., Rybicki L., Khorana A.A. (2017). Identifying predictors for bleeding in hospitalized cancer patients: A cohort study. Thromb. Res..

[6] Pabinger I., van Es N., Heinze G., Posch F., Riedl J., Reitter E.M., Di Nisio M., Cesarman-Maus G., Kraaijpoel N., Zielinski C.C. (2018). A clinical prediction model for cancer-associated venous thromboembolism: A development and validation study in two independent prospective cohorts. Lancet Haematol..

[7] Lee A.Y., Levine M.N., Baker R.I., Bowden C., Kakkar A.K., Prins M., Rickles F.R., Julian J.A., Haley S., Kovacs M.J. (2003). Low-molecular-weight heparin versus a coumarin for the prevention of recurrent venous thromboembolism in patients with cancer. N. Engl. J. Med..

[8] Meyer G., Marjanovic Z., Valcke J., Lorcerie B., Gruel Y., Solal-Celigny P., Le Maignan C., Extra J.M., Cottu P., Farge D. (2002). Comparison of low-molecular-weight heparin and warfarin for the secondary prevention of venous thromboembolism in patients with cancer: A randomized controlled study. Arch. Intern. Med..

[9] Lee A.Y.Y., Kamphuisen P.W., Meyer G., Bauersachs R., Janas M.S., Jarner M.F., Khorana A.A., CATCH Investigators (2015). Tinzaparin vs warfarin for treatment of acute venous thromboembolism in patients with active cancer: A randomized clinical trial. JAMA.

[10] Raskob G.E., van Es N., Verhamme P., Carrier M., Di Nisio M., Garcia D., Grosso M.A., Kakkar A.K., Kovacs M.J., Mercuri M.F. (2018). Edoxaban for the treatment of cancer-associated venous thromboembolism. N. Engl. J. Med..

[11] Young A.M., Marshall A., Thirlwall J., Chapman O., Lokare A., Hill C., Hale D., Dunn J.A., Lyman G.H., Hutchinson C. (2018). Comparison of an oral factor Xa inhibitor with low molecular weight heparin in patients with cancer with venous thromboembolism: Results of a randomized trial (SELECT-D). J. Clin. Oncol. Off. J. Am. Soc. Clin. Oncol..

[12] Agnelli G., Becattini C., Meyer G., Munoz A., Huisman M.V., Connors J.M., Cohen A., Bauersachs R., Brenner B., Torbicki A. (2020). Apixaban for the treatment of venous thromboembolism associated with cancer. N. Engl. J. Med..

[13] McBane R.D., Wysokinski W.E., Le-Rademacher J.G., Zemla T., Ashrani A., Tafur A., Perepu U., Anderson D., Gundabolu K., Kuzma C. (2020). Apixaban and dalteparin in active malignancy-associated venous thromboembolism: The ADAM VTE trial. J. Thromb. Haemost..

[14] Key N.S., Khorana A.A., Kuderer N.M., Bohlke K., Lee A.Y.Y., Arcelus J.I., Wong S.L., Balaban E.P., Flowers C.R., Francis C.W. (2020). Venous thromboembolism prophylaxis and treatment in patients with cancer: ASCO clinical practice guideline update. J. Clin. Oncol. Off. J. Am. Soc. Clin. Oncol..

[15] Kim J.H., Seo S., Kim K.P., Chang H.M., Ryoo B.Y., Yoo C., Jeong J.H., Lee J.L., Im H.S., Jeong H. (2020). Rivaroxaban versus low-molecular-weight heparin for venous thromboembolism in advanced upper gastrointestinal tract and hepatopancreatobiliary cancer. In Vivo.

[16] Schulman S., Kearon C., Subcommittee on Control of Anticoagulation of the Scientific and Standardization Committee of the International Society on Thrombosis and Haemostasis (2005). Definition of major bleeding in clinical investigations of antihemostatic medicinal products in non-surgical patients. J. Thromb. Haemost..

[17] Gordon Lan K.K., DeMets D.L. (1983). Discrete sequential boundaries for clinical trials. Biometrika.

[18] Kraaijpoel N., Di Nisio M., Mulder F.I., van Es N., Beyer-Westendorf J., Carrier M., Garcia D., Grosso M., Kakkar A.K., Mercuri M.F. (2018). Clinical impact of bleeding in cancer-associated venous thromboembolism: Results from the Hokusai VTE cancer study. Thromb. Haemost..

[19] Ay C., Beyer-Westendorf J., Pabinger I. (2019). Treatment of cancer-associated venous thromboembolism in the age of direct oral anticoagulants. Ann. Oncol..

[20] Houghton D.E., Vlazny D.T., Casanegra A.I., Brunton N., Froehling D.A., Meverden R.A., Hodge D.O., Peterson L.G., McBane R.D., Wysokinski W.E. (2021). Bleeding in patients with gastrointestinal cancer compared with nongastrointestinal cancer treated with apixaban, rivaroxaban, or enoxaparin for acute venous thromboembolism. Mayo Clin. Proc..

[21] White R.H., Keenan C.R. (2009). Effects of race and ethnicity on the incidence of venous thromboembolism. Thromb. Res..

[22] Gervaso L., Dave H., Khorana A.A. (2021). Venous and Arterial Thromboembolism in Patients With Cancer: JACC: CardioOncology State-of-the-Art Review. JACC CardioOncol..

[23] Nakamura M., Yamada N., Asamura T., Shiosakai K., Uchino K. (2020). Safety and Effectiveness of Edoxaban in Japanese Venous Thromboembolism Patients―Final Analysis of One-Year Follow-up Data From a Japanese Postmarketing Observational Study (ETNA-VTE-Japan). Circ. Rep..

