# Supplementary material for: A Phase II Study to Compare the Safety and Efficacy of Direct Oral Anticoagulants versus Subcutaneous Dalteparin for Cancer-Associated Venous Thromboembolism in Patients with Advanced Upper Gastrointestinal, Hepatobiliary and Pancreatic Cancer: PRIORITY
Source: Cancers (Basel). 2022 Jan 22;14(3):559. doi: 10.3390/cancers14030559 (PMC8833795; doi:10.3390/cancers14030559)
Supplement: Supplementary file 1 [file cancers-14-00559-s001.zip › cancers-1521724-supplementary.pdf]

A Phase II Study to Compare the Safety and Efficacy of Direct Oral Anticoagulants versus Subcutaneous Dalteparin for Cancer-Associated Venous Thromboembolism in Patients with Advanced Upper Gastrointestinal, Hepatobiliary and Pancreatic Cancer: PRIORITY

Supplementary material

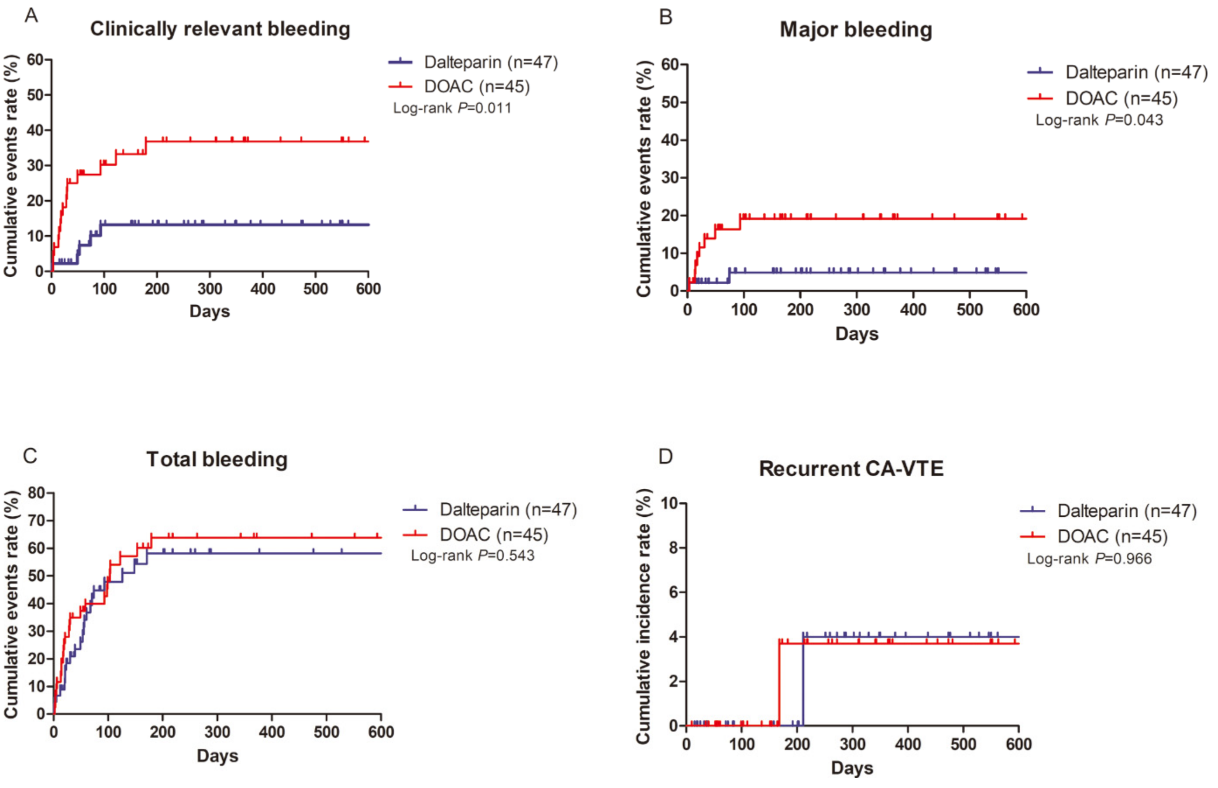

Figure S1. The Kaplan–Meier curves of the time to (**A**) clinically relevant bleeding (major and non-major bleeding), (**B**) major bleeding, (**C**) total bleeding events, and (**D**) recurrent cancer-associated venous thromboembolism in the intent-to-treat set.

Table S1. Definition of recurrence of venous thromboembolism and bleeding.

Recurrence of venous thromboembolism	Major bleeding	Clinically relevant non-major bleeding	Total bleeding
One of the follows: (1) new intraluminal filling defects of venous segment in two or more views on computed tomography (CT); (2) new noncompressible venous segments on ultrasonography; (3) a substantial increase (≥4 mm) in the diameter of the thrombus during full compression in a previously abnormal segment on ultrasonography; (4) new perfusion defect of at least 75% of a segment with corresponding normal ventilation (high probability) on perfusion scan, or (5) new lesions correlated with deep vein thrombosis documented by CT or ultrasonography, despite non–high-probability perfusion defect on perfusion scan; (6) fatal pulmonary embolism based on objective diagnostic tests, which could not be attributed to a documented cause and could not be ruled out (unexplained death).	Acute, clinically overt bleeding accompanied by one or more of the following: contribution to death, occurrence in critical sites such as intracranial site, a decrease in the hemoglobin level of ≥2 g/dL, or the need for the transfusion of ≥2 units of red blood cells	Acute, clinically overt bleeding which did not fulfil the criteria for major bleeding; the need for medical intervention, unscheduled contact with a physician, interruption or discontinuation of anticoagulation, or impairment of activities of daily life	All reported bleeding regardless of the severity

Table S2. Recurrent cancer-associated venous thromboembolism and bleeding events between the rivaroxaban, apixaban, and dalteparin groups.

	Rivaroxaban (*n* = 31, %)	Apixaban (*n* = 13, %)	Dalteparin (*n* = 46, %)	*P** (†/‡/¶)	HR (95% CI)	*P***	HR (95% CI)	*P****	HR (95% CI)	*P*****
Recurrent CA-VTE	1 (3.2)	0 (0)	1 (2.2)	1.000/1.000/1.000	1.56 (0.10-24.93)	0.754	0.03 (0.0-NA)	0.744	0.03 (0.0-NA)	0.681
Category of bleeding events										
Major bleeding	5 (16.1)	3 (23.1)	2 (4.3)	0.078/0.066/0.676	3.89 (0.76-20.08)	0.104	5.28 (0.88-31.64)	0.068	1.31 (0.31-5.49)	0.710
Clinically relevant non-major bleeding	5 (16.1)	3 (23.1)	4 (8.7)	0.472/0.173/0.676	1.84 (0.50-6.86)	0.363	2.80 (0.63-12.51)	0.178	1.51 (0.36-6.31)	0.575
Clinically relevant bleeding	9 (29.0)	6 (46.2)	6 (13.0)	0.082/0.009/0.313	2.37 (0.84-6.66)	0.102	3.93 (1.27-12.22)	0.018	1.58 (0.56-4.44)	0.386
Total bleeding	19 (61.3)	7 (53.8)	23 (50.0)	0.360/1.000/0.742	1.25 (0.68-2.30)	0.467	1.05 (0.45-2.45)	0.911	0.79 (0.33-1.89)	0.597

Abbreviation: CA-VTE, cancer-associated venous thromboembolism; HR, hazard ratio; CI, confidence interval. *† *P* values were estimated using the Chi-square or Fisher`s exact test between rivaroxaban and dalteparin. *‡ *P* values were estimated using the Chi-square or Fisher`s exact test between apixaban and dalteparin. *¶ *P* values were estimated using the Chi-square or Fisher`s exact test between rivaroxaban and apixaban. ** *P* values for HR were estimated using the Cox proportional regression model in rivaroxaban compared to dalteparin.*** *P* values for HR were estimated using the Cox proportional regression model in apixaban compared to dalteparin.**** *P* values for HR were estimated using the Cox proportional regression model in apixaban compared to rivaroxaban.

Table S3. Characteristics of cancer involvement at gastrointestinal mucosa.

	n = 38
Specific sites	
Esophagus	
Upper	0
Middle	4
Lower	3
Esophagogastric junction	0
Stomach	
Cardia	1
Body	9
Antrum	11
Duodenum	7
Ampulla of Vater	2
Colon	1
Endoscopic finding	
Cancer associated mucosal ulceration	25
Cancer stenosis	5
Invasion on adjacent mucosa	8
Endoscopic intervention	
Esophageal stent insertion	1
Gastric stent insertion	3

Table S4. Baseline characteristics in the intent-to-treat set.

	DOAC (n=45)	Dalteparin (*n* = 47)	*P*
Median age, years (range)	63 (39–77)	62 (42–78)	0.542
Sex			0.824
Male	25 (55.6)	24 (51.1)	
Female	20 (44.4)	23 (48.9)	
BMI	22.6 ± 3.3	22.4 ± 3.1	0.838
CA-VTE			0.293
Deep vein thromboembolism	5 (11.1)	11 (23.4)	
Pulmonary thromboembolism	35 (77.8)	32 (68.1)	
Both	5 (11.1)	4 (8.5)	
Tumor types			0.517
Esophageal cancer	7 (15.6)	6 (12.8)	
Gastric cancer	21 (46.6)	18 (38.3)	
Ampulla of Vater cancer	1 (2.2)	1 (2.1)	
Duodenal cancer	0 (0.0)	1 (2.1)	
Hepatocellular carcinoma	2 (4.4)	0 (0.0)	
Biliary cancer	8 (17.8)	9 (19.1)	
Pancreatic cancer	6 (13.3)	12 (25.5)	
ECOG PS			0.661
0–1	38 (84.4)	37 (78.7)	
≥2	7 (15.6)	10 (21.3)	
Metastatic disease	39 (86.7)	34 (72.3)	0.090
Chemotherapy during anticoagulation	41 (91.1)	40 (85.1)	0.375
Lines of chemotherapy during anticoagulation*			0.447
First-line	24 (58.5)	28 (70.0)	
Second-line	12 (29.3)	7 (17.5)	
Third or later line	5 (12.2)	5 (12.5)	
Radiotherapy during anticoagulation	2 (4.4)	1 (2.1)	0.969
Participating sites			
Asan Medical Center	36 (80.0)	38 (80.9)	
Other sites	9 (20.0)	9 (19.1)	

Abbreviation: DOAC, direct oral anticoagulant; BMI, body mass index; GI, gastrointestinal; ECOG PS, Eastern Cooperative Oncology Group Performance Status. *Percentages were calculated among 81 patients treated with chemotherapy during anticoagulation.

Table S5. Recurrent cancer-associated venous thromboembolism and bleeding events in intent-to-treat set **.**

	DOAC (*n* = 45, %)	Dalteparin (*n* = 47, %)	*P**	HR (95% CI)	*P***	Adjusted HR*** (95% CI)	*P***
Recurrent CA-VTE	1 (2.2)	1 (2.1)	1.000	1.06 (0.07–16.98)	0.966	0.97 (0.05–19.23)	0.985
Category of bleeding events							
Major bleeding	8 (17.8)	2 (4.3)	0.048	4.32 (0.92–20.36)	0.064	4.05 (0.86–19.11)	0.077
Clinically relevant non-major bleeding	8 (17.8)	4 (8.5)	0.226	2.11 (0.64–7.02)	0.222	1.66 (0.48–5.71)	0.426
Clinically relevant bleeding	15 (33.3)	6 (12.8)	0.019	2.83 (1.10–7.30)	0.031	2.83 (1.09–7.29)	0.031
Total bleeding	26 (57.8)	23 (48.9)	0.396	1.19 (0.68–2.09)	0.545	1.12 (0.61–2.04)	0.721

Abbreviation: CA-VTE, cancer-associated venous thromboembolism; HR, hazard ratio; CI, confidence interval. * *P* values were estimated using the chi-squared test or Fisher's exact test. ** *P* values were estimated using the Cox proportional regression model. *** Hazard ratio was adjusted with age, sex, and cancer involvement at gastrointestinal mucosa.

Table S6. Univariate analysis for major bleeding and clinically relevant bleeding in intent-to-treat set.

	Major bleeding		Clinically relevant bleeding	
	HR (95% CI)	*P**	HR (95% CI)	*P**
Male vs. Female	2.01 (0.52–7.79)	0.311	1.80 (0.73–4.47)	0.203
Age ≥65 years vs. <65 years	0.28 (0.06–1.30)	0.103	0.55 (0.22–1.37)	0.199
ECOG PS ≥2 vs. ECOG PS 0–1	0.56 (0.07–4.44)	0.584	1.30 (0.44–3.89)	0.636
BMI (<18.5) vs. BMI (≥18.5)	0.90 (0.11–7.20)	0.921	1.33 (0.39–4.54)	0.649
Primary cancer type		0.397		0.245
Upper GI tract cancer	1		1	
Hepatobiliary and pancreas cancer	1.71 (0.49–5.94)		1.67 (0.71–3.94)	
Hemoglobin <9.0 g/dL vs. ≥9.0 g/dL	2.21 (0.57–8.55)	0.252	1.67 (0.61–4.56)	0.321
Platelet <100 × 10^6/μL vs. ≥100 × 10^6/μL	2.37 (0.50–11.17)	0.275	0.94 (0.22–4.02)	0.930
Cr clearance <60 mL/min vs. ≥60 mL/min	1.10 (0.23–5.27)	0.910	1.04 (0.35–3.11)	0.948
Albumin <3.5 g/dL vs. ≥3.5 g/dL	1.87 (0.48–7.25)	0.366	1.69 (0.68–4.19)	0.261
Activated partial thromboplastin time (aPTT)> 35 seconds vs. ≤ 35 seconds	1.14 (0.14–9.00)	0.901	0.51 (0.07–3.84)	0.516
Anticancer systemic therapy during anti-coagulation (yes vs. no)	23.04 (0.0–798399.18)	0.556	23.03 (0.01–36,930.84)	0.405
Treatment lines of systemic therapy during anticoagulation				
First-line	1		1	
Second-line	1.64 (0.39–6.86)	0.499	2.13 (0.81–5.61)	0.127
Third or later line	2.45 (0.47–12.68)	0.286	2.58 (0.80–8.30)	0.111
Radiotherapy during anticoagulation (yes vs. no)	4.07 (0.51–32.17)	0.184	1.66 (0.22–12.37)	0.623
Cancer involvement at GI mucosa (yes vs. no)	2.27 (0.64–8.06)	0.204	2.57 (1.06–6.20)	0.036
Type of anticoagulant (DOAC vs. dalteparin)	4.32 (0.92–20.36)	0.064	2.83 (1.10–7.30)	0.031

Abbreviation: HR, hazard ratio; CI, confidence interval; ECOG PS, Eastern Cooperative Oncology Group Performance Status; BMI, body mass index; Cr, creatinine; GI, gastrointestinal; DOAC, direct oral anticoagulant .
